# A high-throughput model for investigating neuronal function and synaptic transmission in cultured neuronal networks

**DOI:** 10.1038/s41598-017-15171-5

**Published:** 2017-11-03

**Authors:** Jasmeet K. Virdee, Gabriella Saro, Antoine Fouillet, Jeremy Findlay, Filipa Ferreira, Sarah Eversden, Michael J. O’Neill, Joanna Wolak, Daniel Ursu

**Affiliations:** Eli Lilly and Company, Lilly Research Centre, Windlesham, GU20 6PH UK

## Abstract

Loss of synapses or alteration of synaptic activity is associated with cognitive impairment observed in a number of psychiatric and neurological disorders, such as schizophrenia and Alzheimer’s disease. Therefore successful development of *in vitro* methods that can investigate synaptic function in a high-throughput format could be highly impactful for neuroscience drug discovery. We present here the development, characterisation and validation of a novel high-throughput *in vitro* model for assessing neuronal function and synaptic transmission in primary rodent neurons. The novelty of our approach resides in the combination of the electrical field stimulation (EFS) with data acquisition in spatially separated areas of an interconnected neuronal network. We integrated our methodology with state of the art drug discovery instrumentation (FLIPR Tetra) and used selective tool compounds to perform a systematic pharmacological validation of the model. We investigated pharmacological modulators targeting pre- and post-synaptic receptors (AMPA, NMDA, GABA-A, mGluR2/3 receptors and Nav, Cav voltage-gated ion channels) and demonstrated the ability of our model to discriminate and measure synaptic transmission in cultured neuronal networks. Application of the model described here as an unbiased phenotypic screening approach will help with our long term goals of discovering novel therapeutic strategies for treating neurological disorders.

## Introduction

Synapses are essential structural entities of neuronal tissue involved directly in the fast trans-neuronal transmission of information between different parts of the central and/or peripheral nervous system. They are involved in functional and structural plasticity within the complex neuronal circuits found in the brain. Pathological changes in the number and/or turnover of synapses are associated with a number of neurological disorders. In particular, in Alzheimer’s disease (AD) it has been shown that the cognitive decline observed in patients with mild cognitive impairment is correlated with the reduction in the number of synapses^1–5^. Changes related to the synaptic function are often associated with an alteration in activity of receptors expressed at the synapse level. For example, the reduction in synaptic NMDA receptor activity, the so called NMDA receptor hypofunction, is hypothesised to be an important causative mechanism associated with cognitive impairment observed in schizophrenic patients^6,7^.

Various *in vitro* techniques are currently available for studying synaptic function. The classical method for studying synaptic transmission in intact neuronal brain tissue is hippocampal slice electrophysiology. Synaptic transmission in hippocampal slices is very often studied in combination with long term potentiation (LTP) a method used to evaluate also synaptic plasticity^8^. In cultured neuronal networks, whole cell patch-clamp can be used to record miniature post-synaptic excitatory (mEPSCs) or inhibitory (mIPSCs) currents to study indirectly synaptic function combined or not with synaptic plasticity^9^. Though this method doesn’t represent a direct measure of synaptic transmission it offers valuable insights into both pre- and postsynaptic mechanisms involved in synaptic transmission. The above mentioned conventional electrophysiological methods are often low throughput, cost ineffective and are limited in terms of use for large-scale screening in drug discovery.

More recently higher throughput technologies were developed for studying neuronal function in cultured neuronal preparations^10,11^. In one of these models EFS was used to stimulate neuronal activity globally while measuring presynaptic function associated with neurotransmitter vesicle release by means of synapto-pHluorin, a specific genetically encoded fluorescent marker^10^.

Here we present a novel approach for measuring neuronal function and trans-neuronal synaptic transmission in cultured neuronal networks. By using indirect measurements of neuronal activity via calcium flux recordings elicited by EFS and the combination with the state of the art drug discovery instrumentation (FLIPR Tetra), this methodology offers a great potential for performing high-throughput phenotypic screening. We have used selective tool compounds and performed a systematic pharmacological validation of this model by investigating various ion channels and receptors known to be involved in neuronal excitability and synaptic transmission.

## Material and Methods

### Primary neuronal culture

Pregnant (embryonic day 18), time mated, Sprague Dawley (SD) rats were obtained from Charles River (Margate, UK). The pregnant females were killed by CO_2_ followed by cervical dislocation. All animals were treated in accordance with the U.K Animal Scientific Procedures Act of 1986, and all procedures were approved through the British Home Office Inspectorate. Rat cortical neurons (RCN) were prepared from rat embryos as previously published^12^. Triturated rat cells obtained at the end of the dissociation protocol were re-suspended in Neurobasal media containing Neurobrew supplement and 0.5 mM L-glutamine (Invitrogen). The cell suspension was diluted in Neurobrew replacement culture medium for plating cells in 96 well PDL coated plates (Corning, Flintshire, UK) at optimum cell density. After plating, the cells were incubated at 37 °C for up to 12 days *in vitro* (DIV). Media was replenished at DIV 6 and the cells used at DIV 9 or DIV10 for the EFS assay.

### Live cell calcium imaging

**Figure 1:**
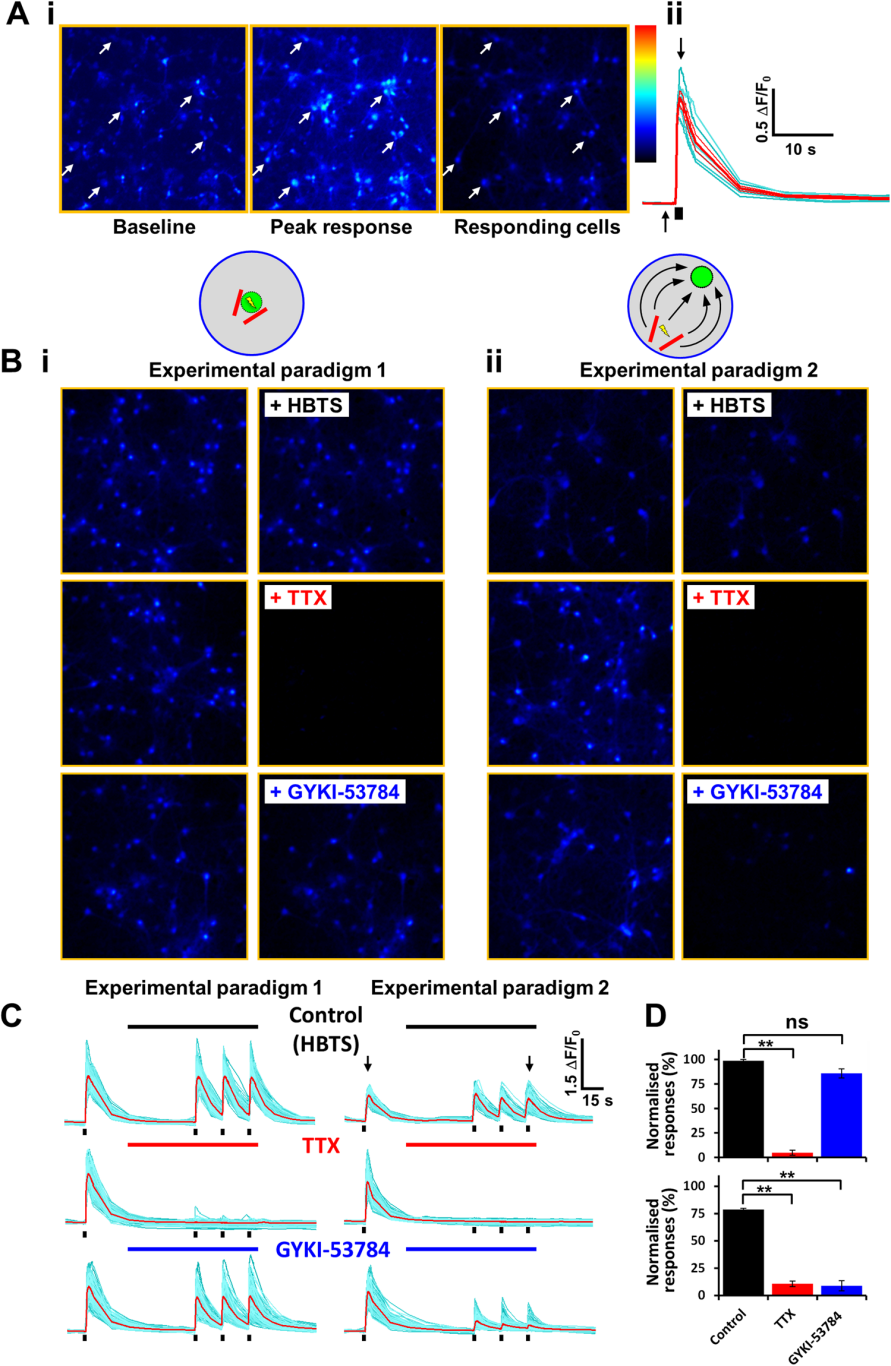
Electrical field stimulation induces synaptically driven calcium responses in primary cultured neurons. (**Ai**) A series of pseudocolour images taken before (baseline) and at the peak (peak response) of the EFS stimulation from a representative live cell imaging experiment. The right hand side panel shows the subtraction of the images thus only displaying the neurones responding to the EFS stimulation. (**Aii**) Cellular responses of the responding neurones corresponding to the white arrows in Ai shown as traces over time. The black arrows denote the time the baseline and peak response images were taken, shown in Ai. The small black bar indicates the EFS train stimulation. (**Bi**) Experimental paradigm 1: The stimulating electrode and field of recording/image acquisition is the same. Representative images (subtracted images) showing calcium responses upon electrical stimulation (10 stimuli, 50 V, 20 Hz) in buffer alone blocked in the presence of TTX (30 nM) but not by GYKI-53784 (10 µM). (**Bii**). Experimental paradigm 2: The stimulating electrode is distal to the field of recording/image acquisition. Stimulation (10 stimuli, 50 V, 20 Hz) in buffer alone shows a robust calcium response, blocked in the presence of TTX (30 nM) or GYKI-53784 (10 µM). (**C**) Individual traces for the experiment shown in Bi and Bii. The small black bars specify the EFS train stimulation. The long black, red, and blue bars represent the time corresponding to the perfusion with HBTS, TTX and GYKI-53784 application, respectively. The black arrows indicate the time the representative images were taken in Bi and Bii. (**D**) A bar chart showing the significant inhibitory effects of TTX and GYKI-53784 on the observed calcium responses, normalised to control (HBTS) in the different experimental paradigms (**p < 0.01). In experimental paradigm 2 the calcium response is blocked by the AMPA antagonist GYKI-53784, thus showing that the observed response is due to synaptic activity.

Rat cortical neurons were loaded with 100 µl of 4 µM of calcium-sensitive dye Fluo4-AM in the presence of 1% pluronic acid (Invitrogen, Paisley, UK) diluted in HEPES buffered Tyrode’s solution (HBTS, Invitrogen, Paisley, UK) containing (mM): 135 NaCl, 5 KCl, 1.2 MgCl_2_, 2.5 CaCl_2_, 10 HEPES, 11 glucose, pH = 7.2. Cells were incubated for 60 min in the dark at room temperature, washed and then subjected to electric field stimulation (EFS) experiments in the presence of HBTS and drugs. Bipolar platinum-iridium electrodes (Science Products GmbH, Hofheim, Germany) were placed adjacent to the optical field (Fig. 1B) and used to deliver voltage pulses with defined characteristics (intensity and frequency) produced by a stimulus generator (NPI, Tamm, Germany). Dye-loaded cells were viewed using an inverted epifluorescence microscope (Axiovert 135TV, Zeiss, Cambridge, UK). Fluo-4 fluorescence was excited by a 480 ± 10 nm light source (Polychrome II, TILL-Photonics, Gräfelfing, Germany) and emission was captured by a iXon 897 EMCCD camera (Andor Technologies, Belfast, UK) after passage through a dichroic mirror (505LP nm) and a high pass barrier filter (515LP nm). A high acquisition frame rate of 10 frames/second was used during application of the EFS pulse train while slower rates were used between recordings (1 frame every 10 seconds). Digitised images were recorded and processed by using Imaging Workbench 5.0 software (INDEC Biosystems, Santa Clara, CA, USA). Data were analysed by averaging individual traces collected from a large number of cells in multiple wells of the 96-well plate. Delta F/F0 values were measured by calculating the ratio between the change in fluorescence signal intensity (delta F) and baseline fluorescence (F0).

### Measurement of Ca^2+^ flux using FLIPR Tetra

Media was removed and cells were incubated in HBTS assay buffer (10 mM HEPES, 135 mM NaCl, 5 mM KCl, 2.5 mM CaCl_2_, 1.2 mM MgCl_2_, 10 mM glucose) in the presence of 4 μM Fluo 4-AM and 0.05% pluronic F127 (Invitrogen) for 1 h in the dark. The solution was then replaced with assay buffer immediately before transferring the plate onto the FLIPR Tetra (Molecular Devices, UK). A drug plate was prepared before running the EFS assay, typically containing a serial dilution of the tested compound. The EFS was run once, then drug solution was added and 3 minutes later the EFS was run once again. Fluorescence signals were recorded at a rate of 3 samples per seconds with a 0.25 s exposure time.

**Figure 2:**
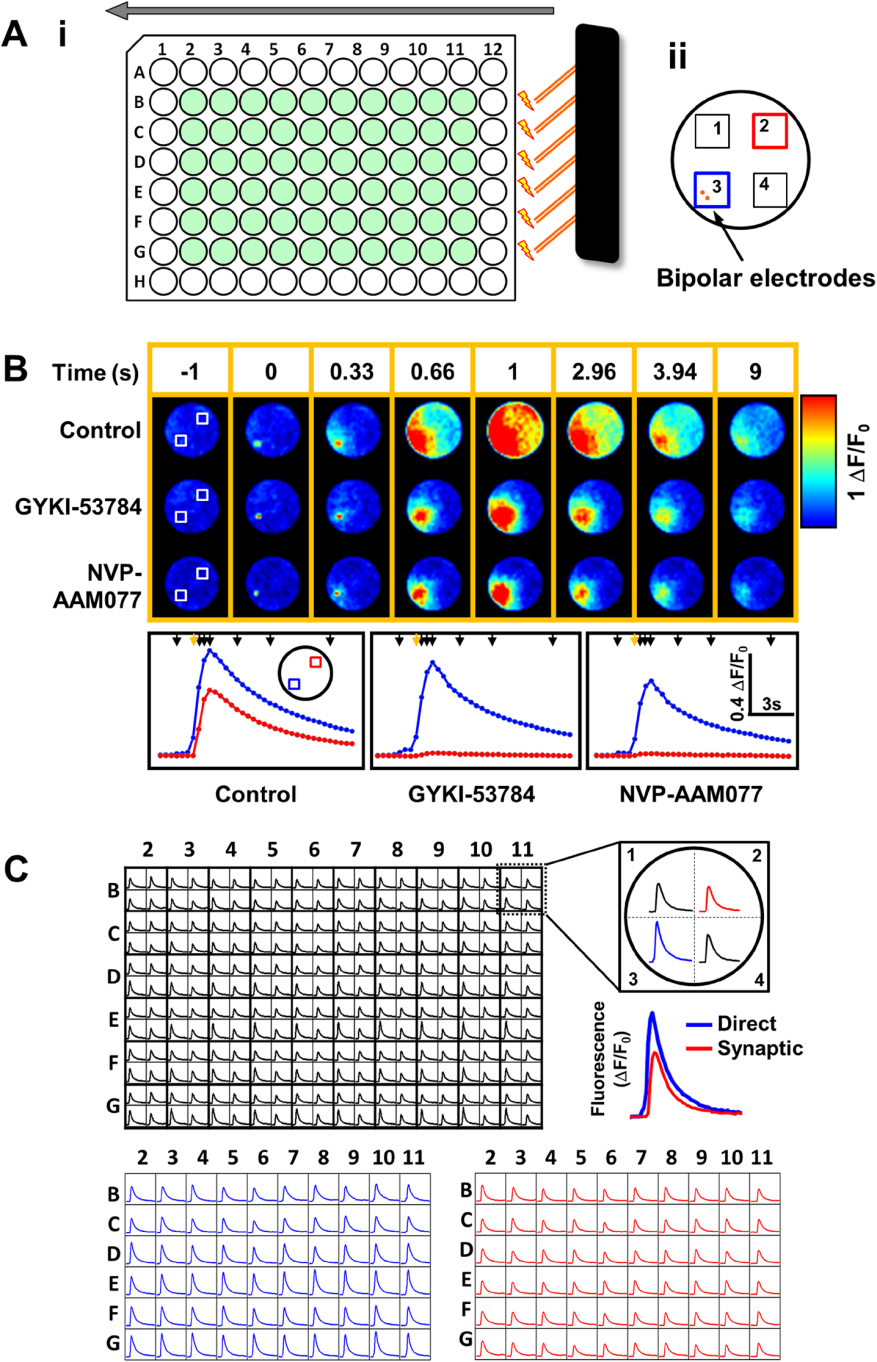
Recording EFS-evoked neuronal and synaptic responses by using the FLIPR Tetra system. (**Ai**) A representative cartoon of the experimental system. 6 bipolar electrodes are placed within the FLIPR Tetra and moved across the 96 well plate by a motorised linear actuator (direction indicated by the arrow) and stimulate only those wells coloured in green (excluding edges). (**Aii**) Data are obtained from four individual points in a single well. The bipolar electrodes are confined to one of the four regions, region 3, shown by the blue square. (**B**) Representative pseudocolour images from a 96 well plate showing the changes in calcium concentration during the course of a typical experiment. At time 1 s the calcium concentration is at its peak in the control well. The traces corresponding to the pseudocolour images are shown in the lower panel. The black arrows at the top correspond to the images shown in the top panel; the orange arrow indicate the time the EFS was applied. (**C**) A typical plate readout from a 96 well plate, presented as a 384 well plate (stimulation protocol, 25 Hz, 10 stimuli, 25 Volts). For a single well there are 4 individual minigraphs plots, shown to the right. Quadrant 3 represents a direct response (shown in blue, proximal to the electrode), and Quadrant 2 represents a synaptic response (shown in red, distal to the electrode). Below there are typical plate readouts of only the direct response (shown in blue, 60 individual readouts) and readouts of only the synaptic response (shown in red, 60 individual readouts).

A set of 6 bipolar platinum-iridium electrodes (Science Products GmbH, Hofheim, Germany) were assembled on a custom built holder attached to a dual motorized linear actuator (Zaber Technologies, Vancouver, Canada) that allowed software-controlled movement of the electrodes across the length of the plate and micro-positioning inside each well (see Fig. 2A). A stimulus generator (NPI, Tamm, Germany) was used to deliver defined electrical field stimulation protocols directly into the wells in the 96 well plate. Customised software was used to analyse and detect maximum fluorescence amplitude in the signal recorded following electrical stimulation in individual wells. The maximum fluorescence was measured before and post drug delivery and compared. Data were then normalised to give a normalised response compared to vehicle control wells. To obtain IC_50_ values, data points were fitted to a 4-parameter logistic curve fit model using GraphPad Prism software (San Diego, CA). IC_50_ values are presented as mean and 95% confidence intervals (in text and in Table 1).

**Table 1:** Effects of different classes of neuronal/synaptic modulators on EFS evoked calcium responses. Least-square fit data for IC50, Hill slope (h) and 95% confidence intervals (CI) are presented for direct and synaptic responses; n (number of experiments), replicates (total number of individual wells, from independent experiments); nf (no fit – data could not be fitted).

Drug	Direct response			Synaptic response			n (replicates)
	IC_50_ (nM)	h	95% CI	IC_50_ (nM)	h	95% CI	
TTX	3.2	1.34	2.4 to 4.0	0.5	1.47	0.3 to 0.8	3 (10)
Lamotrigene	7985	1.6	5723 to 11140	1813	1.3	971 to 3385	3 (7)
Retigabine	37820	1.18	24540 to 58290	1325	0.75	918 to 1911	3 (14)
GYKI-53784	nf	nf	nf	365.3	2.9	276 to 483	3 (10)
NBQX	nf	nf	nf	19.04	1.24	9 to 37	3 (14)
NVP-AAM077	nf	nf	nf	174.0	1.5	121 to 249	3 (12–18)
Bicuculine	nf	nf	nf	1810	1.86	937 to 3492	3 (10)
Gabazine	nf	nf	nf	270	3.1	126 to 577	3 (10)
ω-connotoxin GVIA	123.5	1.3	48.5 to 314.2	42.9	1.75	26.1 to 70.8	3 (7)
ω-agatoxin IVA	52.2	1.8	16.3 to 167.3	37.5	3.95	22.1 to 63.8	3 (7)
LY379268	2.54	1.4	0.8 to 7.1	1.54	1.4	0.8 to 2.6	5 (36)

### Immunocytochemistry

Neurons were fixed with 4% paraformaldehyde (Affymetrix, UK) for 45 min at RT and then permeabilised and blocked with PBS-0.1% Triton × 100 containing 5% BSA for 1 h at RT. Antibodies directed against Synapsin1 (SYSY, 106011, used at 1/500 dilution) and MAP2 (Abcam, AB5392, used at 1/10000 dilution) were diluted in PBS with 0.1% triton × 100 and 0.5% BSA and added to the cortical neurons overnight at 4 °C. Cells were incubated with Alexa-488 anti-mouse and Alexa-555 anti-chicken (Invitrogen, both used at a 1/1000 dilution) in PBS with 0.1% triton × 100 and 0.5%BSA for 1:30 hours at RT in the dark. Nuclei were counterstained with Hoechst at 10 µg/ml (Life technologies, UK). Fluorescent images were recorded on a FV1000 Olympus confocal microscope. Image files were analysed by using Cell Profiler^13^. Briefly images were segmented and objects were detected in the Synapsin 1 channel.

### Statistical analysis

Statistical analysis was performed by using Prism 5 (GraphPad Software Inc). Student’s two-sided t-test was used to test for significant differences of mean values (assuming two independent populations; p = 0.05 unless otherwise stated, Fig. 1D). For the time dependent changes in synaptic connectivity experiment statistical significance was assessed by using a two way ANOVA analysis.

### Data Availability Statement

All data generated or analysed during this study are included in this published article.

## Results

### Electrical field stimulation induces synaptically driven calcium responses in primary cultured neurons

As part of our ongoing efforts to develop new cellular models for assessing pharmacology of native neuronal receptors we carried out a series of experiments that explored the use of electrical field stimulation (EFS) for induction of neuronal activity. This form of triggering neuronal activity which relies on induction of action potentials (APs) following application of an external electrical stimulus resembles the physiological form of neuronal activation (ie synaptic initiation of APs). We used an indirect methodology for recording neuronal activity by measuring the intracellular changes in calcium concentration via a calcium sensitive dye. For all our experiments we have used rat embryonic cortical neurons plated at optimum density in multi-well plates (96 well format) and cultured for variable durations (5 to 12 DIV).

Our preliminary experiments were performed by using a standard live cell imaging setup that allowed for semi-automatic recording from multi-well plates. The system has the capability to acquire fluorescent images from individual wells of the plate whilst continuously perfusing with buffer/compounds. Electrical stimulation can be applied through a pair of platinum-iridium electrodes placed adjacent to the image recording field.

A typical calcium imaging experiment is shown in Fig. 1A. A stimulus train with defined parameters (10 stimuli, 50 V, 20 Hz, 2 ms stimulus duration) was applied to the cultured rat cortical neurons (9 DIV) loaded with the calcium dye Fluo-4 AM and fluorescent images were recorded at short intervals (10 frames/second). Fig. 1Ai shows a series of pseudocolor images, one recorded before EFS application (baseline image), an image collected at the maximum calcium response (peak response at 2 seconds after EFS), and an image that represents the subtraction between the peak response and baseline image, respectively. The subtracted image allows one to visualise only the cells that respond to the electrical stimulation.

Following a segmentation analysis based on a simple thresholding algorithm the areas corresponding to single neurons were identified and used to extract the timecourse of individual cellular responses. Traces corresponding to highlighted neurons (white arrows) are shown in Fig Aii. This experiment demonstrates that we are able to evoke and record robust calcium responses following application of EFS in rat cortical neurons.

We used this approach to investigate the effects of selective pharmacological tools on modulating EFS evoked responses in cultured neurons. One of the aims of this study was to explore whether we can use the EFS protocols to induce and measure synaptic driven responses in cultured rat cortical neurons. For this experiment we plated cortical neurons at an optimum density (0.6 × 10^5^ cells/well) that is known, based on our previous studies^14^, to promote neuronal network formation and synaptic connectivity. We performed this set of experiments under two stimulation paradigms. First we positioned the field electrodes in the middle of the well directly above the area used for image acquisition (Fig. 1Bi, Experimental paradigm 1). This is similar to the experiment presented in Fig. 1A. For the second paradigm we positioned the field electrodes towards the edge of the well while recording images from the opposite side of the well (Fig. 1Bii, Experimental paradigm 2). The hypothesis driving this second protocol was that while stimulating neuronal activity on one side of the well the excitation will propagate via synaptic transmission to the whole neuronal network in the well and will reach the area from where we acquire images.

We combined the above described stimulation paradigms with the assessment of effects of selective pharmacological tools on the EFS induced responses. After the first EFS stimulus (10 stimuli, 50 V, 20 Hz) the cells were perfused with control buffer (HBTS) with/without compounds for 1 min followed by application of 3 similar EFS trains at 15 sec intervals. Figure 1B shows representative images (baseline subtracted images, 2 sec after EFS) for the two stimulation paradigms, before and after perfusion with buffer alone or buffer containing compounds. Individual traces corresponding to the images in Fig. 1B are shown in Fig. 1C with the averaged data from n = 3 replicates in Fig. 1D. For illustrating the compound effects we are showing the image corresponding to the 3^rd^ peak response (as indicated by the black arrows in Fig. 1C).

One can observe that under control conditions (perfusion with buffer alone top panels; Fig. 1Bi and Bii) we were able to elicit reproducible EFS calcium responses. Importantly, we also observed robust EFS responses under the condition where the electrodes were placed away from the image recording area (Experimental paradigm 2; Fig. 1Bii). Neurones were subsequently perfused with 30 nM tetrodotoxin (TTX), a selective sodium channel blocker, and in both experimental paradigms the direct stimulation response and those elicited further away from the electrodes were completely blocked by the compound (centre panels in Fig. 1Bi and Bii). This study demonstrates that EFS calcium responses are evoked via induction of APs and not caused by membrane disruption artefacts induced by the electrical stimulus.

Following this study we used a selective non-competitive AMPA receptor antagonist, GYKI-53784^15^ to probe whether the responses elicited by EFS are modulated by AMPA receptors. AMPA receptors are known to be the major postsynaptic ionotropic glutamate receptors that mediate fast synaptic transmission in the central nervous system. Application of 10 µM GYKI-53784 within Experimental paradigm 1 had no effect on the calcium responses triggered by direct stimulation. However, in Experimental Paradigm 2 (stimulation area away from the image recording area) application of 10 µM GYKI-53784 blocked the responses completely. These results demonstrated that blocking of AMPA receptors inhibited the propagation of excitation from the region under the electrodes (area of stimulation) to the region where the images are recorded.

We can conclude from this set of experiments that we can evoke and measure synaptic transmission in primary neuronal networks.

### Recording EFS-evoked neuronal and synaptic responses by using the FLIPR Tetra system

While the above methodology is performed with a semi-automated system where the software controls well-to-well movement, drug perfusion and electrical stimulation it still represents a relatively slow process that requires relatively lengthy recordings.

The advantage of using the calcium flux assay for measuring neuronal activity is represented by the possibility of transferring any developed assay to higher-throughput cellular screening instruments which are readily available in the drug discovery environment (FLIPR®, Molecular Devices, US, FDSS®, Hamamatsu, Japan). Our subsequent goal was to explore whether we could use the FLIPR Tetra system to perform similar type of recordings as described above in the single cell calcium imaging system. The FLIPR Tetra can record kinetic fluorescent signals from multi-well plates while having a fully integrated liquid handling system that allows addition of drug solutions during data recording.

We modified the system and added a set of 6 platinum-iridium bipolar electrodes placed on a motorised dual linear actuator. This allows movement across the length of the plate at the same time with micro-positioning of the electrodes inside each well (Fig. 2A). In our multi-well plate experiments we usually do not use the full area of the plate but only the inner wells (shown in green, Fig. 2A) because of the common edge effects associated with culturing cells for longer duration (up to DIV14 in our study).

In order to replicate the live cell imaging experiment above (Fig. 1Bii), which demonstrated the possibility to record synaptic transmission in a single well, we had to enable the ability to record in parallel from different areas of the same well on the FLIPR. The FLIPR system is a simple live cell imaging system that can acquire fluorescent images over time. Because the recorded images cover the full area of the plate they are usually low resolution so one cannot really identify individual cells. Conventionally, the FLIPR system reports data by calculating an average of a region of interest in the center of the well. A simple way to enable multiple recording points from the same well is by switching the recording mode from a 96 well format to a 384 well format. While still recording from a 96 well plate the system will now report data obtained from 4 regions of interest in a single well. The regions can be positioned as outlined in Fig. 2Aii. We positioned the stimulating electrodes in region 3 of an individual well. In this configuration we can record in parallel the kinetic fluorescent signal under the stimulation electrodes (ie region 3) as well as further away from the electrodes (ie region 1, 2 and 4).

Firstly, on the FLIPR system we wanted to confirm the data obtained above in the single cell imaging assay. We maintained all the assay conditions, same cell density (0.6 × 10^5^cells/well), time in culture (DIV 9) and stimulation parameters (10 stimuli, 50 V, 20 Hz, 2 ms stimulus duration) and combined the study with the evaluation of two selective compounds targeting synaptic receptors: GYKI-53784 (an AMPA receptor antagonist) and NVP-AAM077 (an NMDA receptor antagonist). Figure 2B shows pseudocolor images outlining changes in calcium concentration during the timecourse of the experiment. Images of representative wells from a 96 well plate are shown corresponding to the 3 different conditions used in this experiment: Control, 10 µM GYKI-53784 and 10 µM NVP-AAM077. Image data was acquired at a framerate of 3 images/second and electrical stimulation was applied at time 0. The traces shown in the lower panel of Fig. 2B were measured from the rectangular regions highlighted in white in the images corresponding to time −1. These are the regions 2 and 3 as outlined in Fig. 2Aii. At the time immediately after stimulation one can observe a rise in the calcium level directly under the electrodes (t = 0.33 sec). The calcium response propagates to neurons adjacent to the stimulation area and reaches the distal region of interest in the control well condition. However, in the wells with cells incubated with the AMPA and NMDA receptor antagonists one could observe that the first calcium response appears in the proximal region but doesn’t propagate beyond the middle of the well, therefore no calcium response could be recorded in the distal region. The traces corresponding to the experiment described above are shown in Fig. 2B, lower panel. Both compounds blocked transmission of excitation throughout the neuronal network in the well while the amplitude of the responses recorded from neurons placed under the electrode was similar to the control condition.

The results obtained here confirmed the observations from the single cell live cell imaging assay and provided in addition the means to record in parallel two spatially separated signals. The first response, recorded in the neurons localised under the electrodes, corresponding to direct induction of neuronal excitability by the electrical field stimulation, will be referred to from now on as the direct response. The second response, recorded further away from the electrode, corresponding to the trans-neuronal propagation of the excitation via synaptic transmission, will be referred to from now on as the synaptic response.

Figure 2C shows a representative plate readout from a typical experiment. The first layout shows the direct output of the system as a 384 kinetic minigraphs plot with 4 readouts for each well as explained above and outlined again in the right hand side inset. For all the experiments presented throughout the rest of the manuscript we have used only the data collected from region 3 (direct response – blue line) and region 2 (synaptic response – red trace). To illustrate the typical data the assay can provide we have extracted in the lower panel of Fig. 2C, in two separate plate layouts, the data from the direct (left – blue traces) and synaptic (right-red traces) responses.

**Figure 3:**
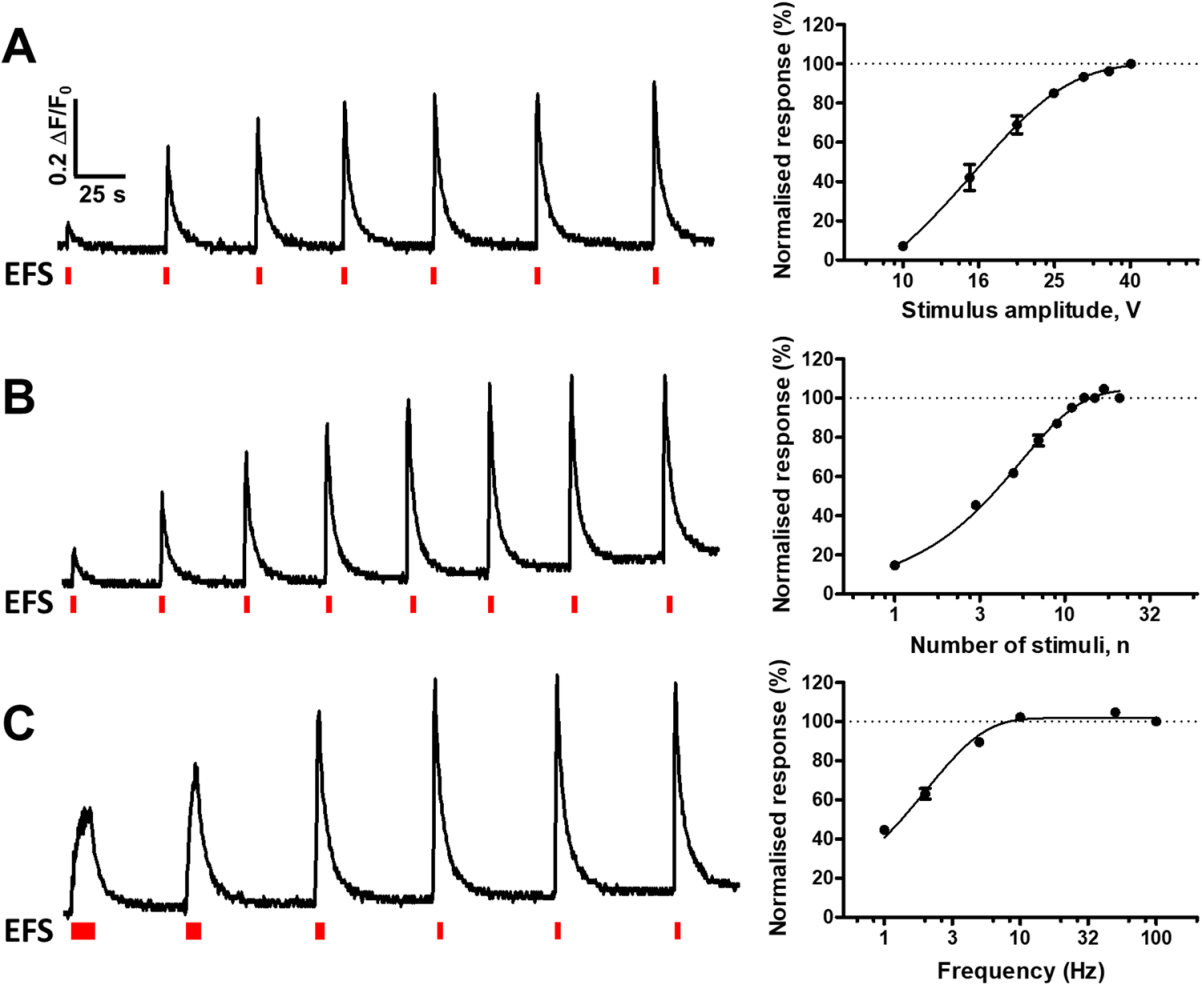
Modulation of neuronal responses by changes in stimulation parameters. All experiments were run in the presence of 10 µM GYKI-53784 and 10 µM NVP-AAM077. (**A**) Increasing the voltage amplitude results in an increase in the calcium response (stimuli 10, 25 Hz). (**B**) Increasing the stimuli number (20 V, 25 Hz) increases the amplitude of the calcium response. Note that a single stimulus can elicit a calcium response. (**C**) Changes in frequency stimulation (10 stimuli, 20 V) show a slow and steady increase in calcium responses.

Thus far we have demonstrated the overall principle of the methodology. We now move on to the optimisation and characterisation of the various assay conditions. Figure 3 shows data generated from experiments where repeated stimulation was applied in the same well to study relationship between calcium response amplitude and the EFS amplitude, frequency and number of stimuli. These experiments were run in the presence of AMPA and NMDA receptor antagonists (10 µM GYKI-53784 and 10 µM NVP-AAM077) in order to exclude any potential contribution of the synaptic component to the direct responses recorded here. We observed a clear correlation between the EFS amplitude and the calcium response amplitude (Fig. 3A). The protocol was run at constant number of stimuli (10) and stimulation frequency (25 Hz). While increasing the amplitude of the electrical stimulus, more and more cells are recruited and respond to stimulation, hence the increased calcium response amplitude. A similar relationship was observed with increasing the number of stimuli in the EFS train (run at 20 V, 25 Hz) Fig. 3B. Each stimulus corresponds theoretically to one AP potential being generated in a neuron which upon activation of voltage-gated Ca^2+^ channels would cause influx of calcium into the cell. The more APs are triggered by the EFS train the larger the calcium response will be. It is important to highlight here that the system is sensitive enough to measure a calcium response triggered by a single stimulus, ie a single AP. Finally, changing the interval between the stimuli can also affect the amplitude of the calcium response (Fig. 3C). At lower stimulation frequencies (ie 1 Hz) responses were smaller because there was enough time between two consecutive stimuli to allow for recovery of intracellular calcium concentration by transport either into endoplasmic reticulum or to the extracellular space. Higher stimulation frequency will cause a rapid influx of calcium upon consecutive APs hence the higher response amplitude. Based on these results we have selected 10 stimuli, 20 V and 25 Hz as the default stimulation parameters for all the subsequent recordings.

### Time dependent changes in synapse formation and synaptic connectivity

**Figure 4:**
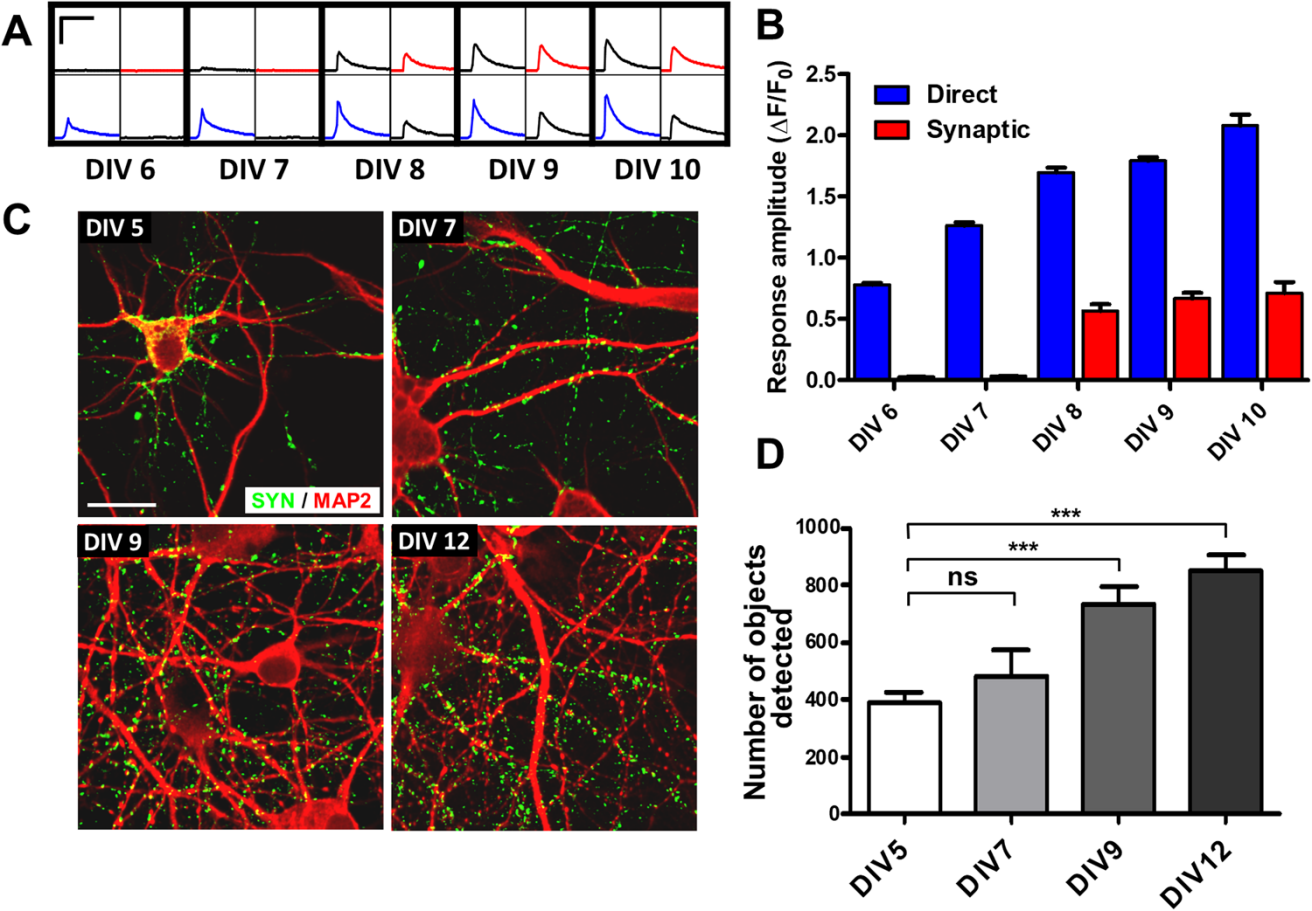
Synaptic connectivity and synapse number increase over time in culture. (**A**) Example wells from different DIV, showing the changes in direct and synaptic responses over time (10 stimuli, 20 V, 25 Hz). The scale bars correspond to 1 ΔF/F_0_ and 10 sec. (**B**) Bar chart showing average data for the increase in calcium response over DIV (n = 10 wells per condition). There was a significant increase (p < 0.001) in the amplitude of evoked calcium transients over time in culture and between the direct and synaptic responses amplitude. (**C**) Representative confocal images of rat cortical neurones immunolabelled at different times in culture. Neurones are labelled with MAP2 (red) to detect the dendrite specific microtubule protein, and synapsin 1 (green) to detect the presynaptic regions; Scale bar is 25 µm. (**D**) Quantification of the synapse number shows a significant increase over DIV. (***p < 0.001).

We have used the above methodology to characterise the neuronal network development and synaptic connectivity in cultured rat cortical neurons. We plated rat cortical neurons at a density of 0.6 × 10^5^cells/well and maintained them in culture for different lengths of time (6, 7, 8, 9 and 10 days) followed by recording of EFS evoked responses in the FLIPR assay. Representative single well recordings (4 analysed regions) are shown for the different days in culture in Fig. 4A. One can observe a robust increase (almost 2x fold) in the direct response (blue traces) with longer times in culture. The synaptic responses (red traces) are absent during the earlier times in culture (DIV6 and DIV7) but appear after 8 days in culture. The amplitude of direct and synaptic responses were analysed for multiple wells (n = 6) and are plotted in Fig. 4B as averaged data. Both the time dependent changes and the direct vs synaptic responses amplitude were found to be highly significant (p < 0.001) when analysed by two-way Anova.

In parallel with the experiment described above we performed a study aimed to analyse, by using immunocytochemistry, synapse formation during a similar time course in culture. We have used specific antibodies for a presynaptic protein (Synapsin) and for a dendrite specific microtubule protein (MAP2) (Fig. 4C) and labelled rat cortical neurons after 5, 7, 9 and 12 days in culture. The number of synapses was quantified as number of objects detected in the synapsin channel (green channel in Fig. 4C) present in the close proximity to the MAP2 staining (dendrites – red channel in Fig. 4C). Analysed data from multiple wells (3 wells, 2 images/well) are plotted in Fig. 4D. One can observe a significant increase in the number of synapsin labelled objects over the time course of the experiments reaching a maximum level at DIV 12.

These experiments show that there is an optimum synaptic density that is achieved over time in culture that enables propagation of excitation throughout the whole network.

### EFS evoked neuronal responses are modulated by known pharmacological tools

We have shown in Figs 1 and 2 that the calcium responses evoked by EFS in cultured rat cortical neurons can be blocked by known ion channel antagonists (TTX, GYKI-53784 and NVP-AAM077). In the subsequent set of experiments we performed a thorough pharmacological characterisation of the FLIPR EFS assay by evaluating effects of various tool compounds, reported in the literature as selective modulators of neuronal/synaptic activity. We focused on three different classes of drugs: i) compounds that modulate ion channels involved in neuronal excitability (sodium and potassium channels), ii) compounds that modulate post-synaptic receptors (AMPA, NMDA and GABA-A receptors) and iii) compounds that modulate pre-synaptic ion channels and receptors involved in neurotransmitter release, voltage gated calcium channels (VGCCs) and mGluR2/3 receptors.

**Figure 5:**
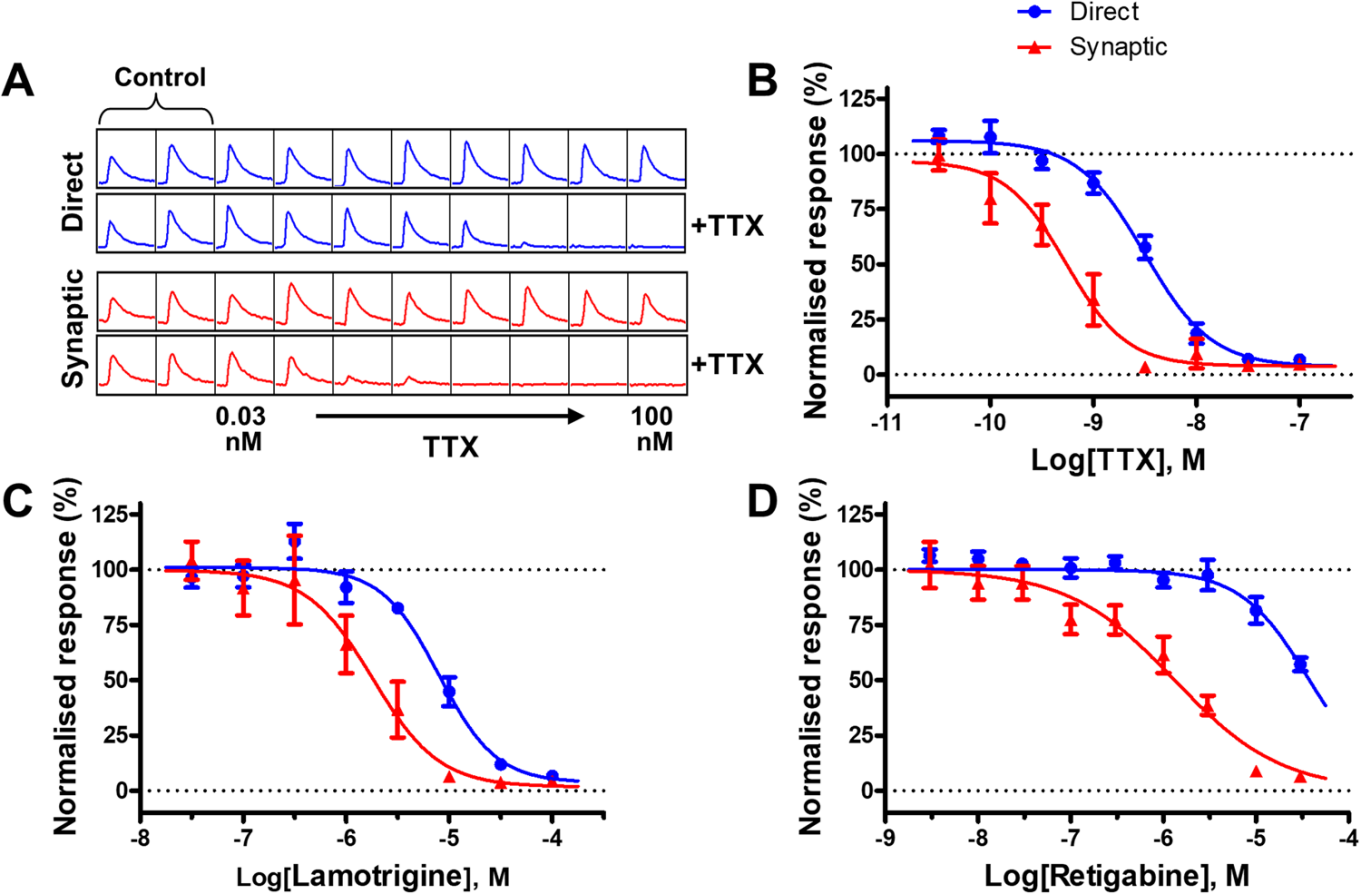
Modulation of EFS evoked calcium responses by compounds influencing neuronal excitability. (**A**) Representative traces showing the direct (blue traces) and synaptic responses (red traces; 10 stimuli, 20 V, 25 Hz) before and post the application of TTX at varying concentrations. Note the decrease in amplitude of both direct and synaptic responses upon increasing concentration of TTX. The data are plotted and shown in (**B)** with an IC_50_ value of 3.2 nM for the direct responses and 0.5 nM for the synaptic responses. (**C**) The application of lamotrigene resulted in a dose dependent decrease in both the direct and synaptic responses, with an IC_50_ value of 7.9 µM and 1.8 µM, respectively. (**D**) Increasing concentrations of retigabine, a selective potassium channel opener, caused inhibition in the direct and synaptic with a potency of 37.8 µM and 1.32 µM, respectively.

Figure 5A shows representative traces from an experiment designed to measure the concentration response curve for a selective sodium channel blocker, TTX. The EFS assay was run once to establish the baseline response (top traces for the direct and synaptic responses, Fig. 5A), followed by the addition and incubation (3 min) of the test drug at different concentrations. A second EFS run was recorded in order to assess the effects of the drug (bottom traces for the direct and synaptic responses in Fig. 5A). Both the direct and synaptic responses were blocked by TTX with an IC_50_ value of 3.2 nM for the direct responses and 0.5 nM for the synaptic response, respectively (Fig. 5B, see also additional least-square fit data in Table 1). The same protocol was used to assess the activity of an additional sodium channel blocker, lamotrigene (Fig. 5C). Lamotrigine blocked the EFS responses with an IC_50_ value of 7.9 µM for the direct responses and 1.8 µM for the synaptic response, respectively. We next evaluated retigabine, a subtype selective potassium channel opener^16^. Retigabine activates mainly heteromeric Kv7.2 and Kv7.3 channels and as a result causes changes in resting membrane potential towards more negative levels (ie hyperpolarization). The immediate effect is a reduction in neuronal excitability, by reducing the generation and propagation of action potentials. The data we obtained in the EFS assay shows a clear effect of retigabine on inhibiting EFS evoked responses (Fig. 5D) with a potency of 37.8 µM on direct responses and 1.32 µM on synaptic responses.

**Figure 6:**
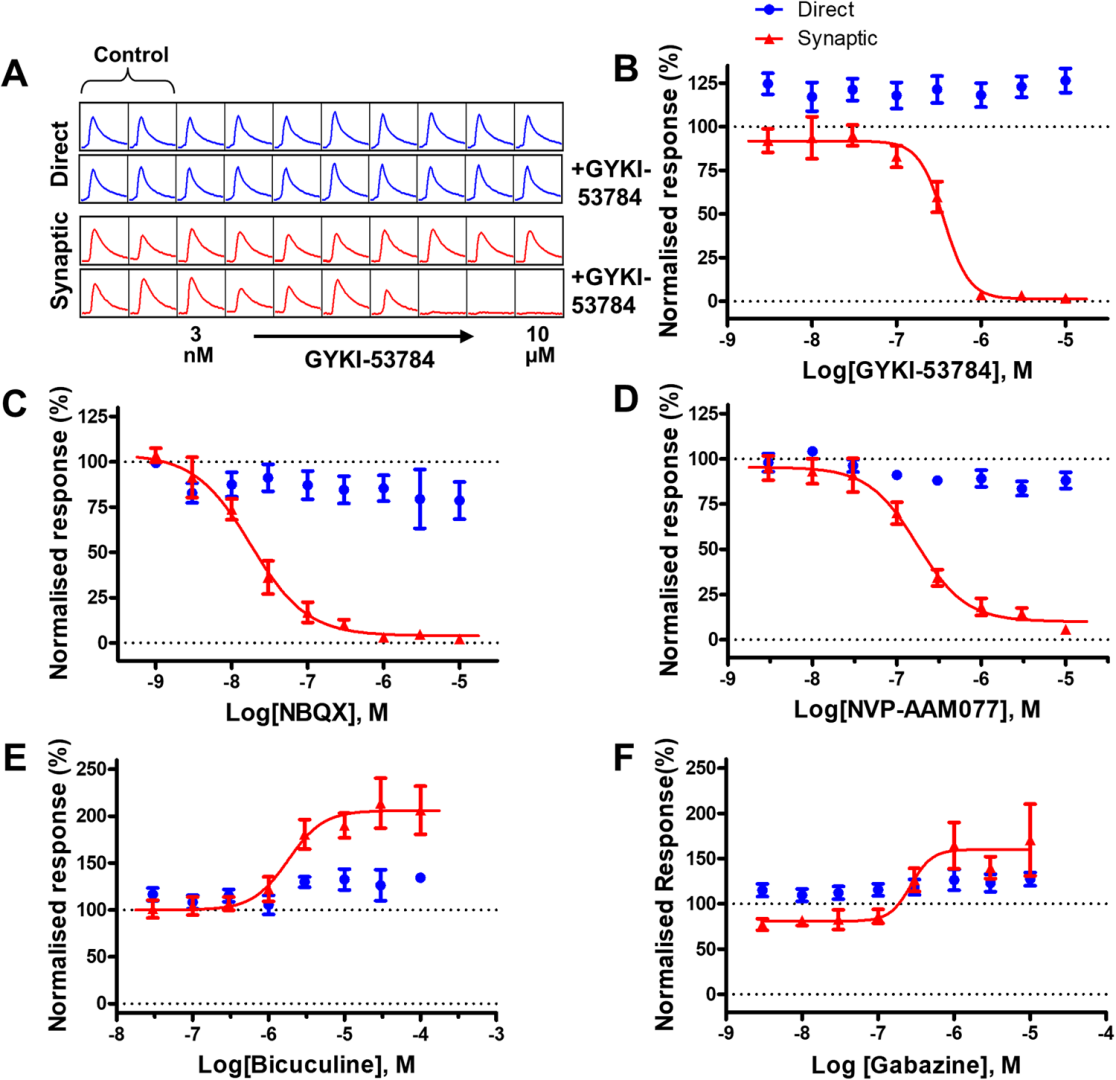
Modulation of EFS evoked calcium responses by compounds acting at postsynaptic receptors. (**A**) Representative traces showing the direct (blue traces) and synaptic responses (red traces; 10 stimuli, 20 V, 25 Hz) before and post the application of GYKI-53784, a selective non-competitive AMPA receptor. Note the steady decrease in amplitude of the synaptic response upon increasing concentrations of GYKI-53784 with no effect on the direct responses (blue). This is plotted and shown in (**B**) with an IC_50_ value of 0.36 µM for the synaptic responses. (**C**) Application of NBQX, a non-NMDA antagonist, caused a dose dependent decrease in the synaptic response, with IC_50_ of 19.04 nM. Note that there is no effect on the direct response (blue trace) (**D**) Normalised CRC showing that NVP-AAM007, a selective competitive NMDA antagonist, blocks synaptic responses with an IC_50_ of 174 nM with no effect on the direct response. The competitive GABA-A antagonists bicuculline (**E**) and gabazine (**F**) caused a concentration dependent increase in the amplitude of the synaptic response with IC_50_ values of 1.81 µM and 0.27 µM, respectively. Note the minimal effects observed on the direct responses.

Subsequently we focused on investigating receptors expressed at synapse level known to be directly involved in physiological synaptic transmission. Firstly, we evaluated pharmacological tools that block post-synaptic receptors (Fig. 6). GYKI-53784 (an AMPA receptor antagonist) has been shown to block AMPA-mediated excitotoxicity in cultured hippocampal neurons with an IC_50_ of 2 µM^17^. We confirmed here the data we obtained in the single concentration experiments with GYKI-53784 (Figs 1 and 2) and showed a concentration dependent block of synaptic transmission with an IC_50_ of 0.36 µM (Fig. 6B). GYKI-53784 had no effect on EFS evoked direct responses. Representative traces for this experiment are shown in Fig. 6A, before and after drug addition and illustrates the specific effect of the compound on the synaptic function and lack of effects on neuronal excitability. We tested further an non-NMDA antagonist in our assay, NBQX, reported to be active at both AMPA receptors (between 30 and 300 nM)^18,19^, and KA receptors (between 80 nM and 3 µM)^20,21^. NBQX blocked the synaptic responses in our assay with an IC_50_ of 19.04 nM but had no effect on direct responses, following a similar pattern we have seen with the other AMPA receptor antagonist tested here (GYKI-53784).

We continued our evaluation and tested the involvement of another ionotropic glutamate receptor (NMDA receptor) in the EFS evoked calcium responses. NVP-AAM007 is a selective competitive NMDA antagonist with specificity for NR1/NR2A containing receptors vs NR1/NR2B receptors^22^. NVP-AAM007 caused a concentration dependent block of synaptic responses with an IC_50_ of 174 nM but had no effect on the direct response component (Fig. 6D). We then went on to evaluate the role of GABA-A receptors in modulating the calcium responses recorded in the EFS assay. GABA-A represent the major inhibitory neurotransmitter receptors so theoretically blocking their activation in a neuronal network should remove the tonic inhibition and cause enhancement of evoked responses. We tested here bicuculine and gabazine, two GABA-A competitive antagonists and observed indeed a concentration dependent increase in the amplitude of synaptic responses (IC_50_ = 1.81 µM for bicuculine and 0.27 µM for gabazine) whilst only minimal effects were observed on the direct responses (Fig. 6E).

**Figure 7:**
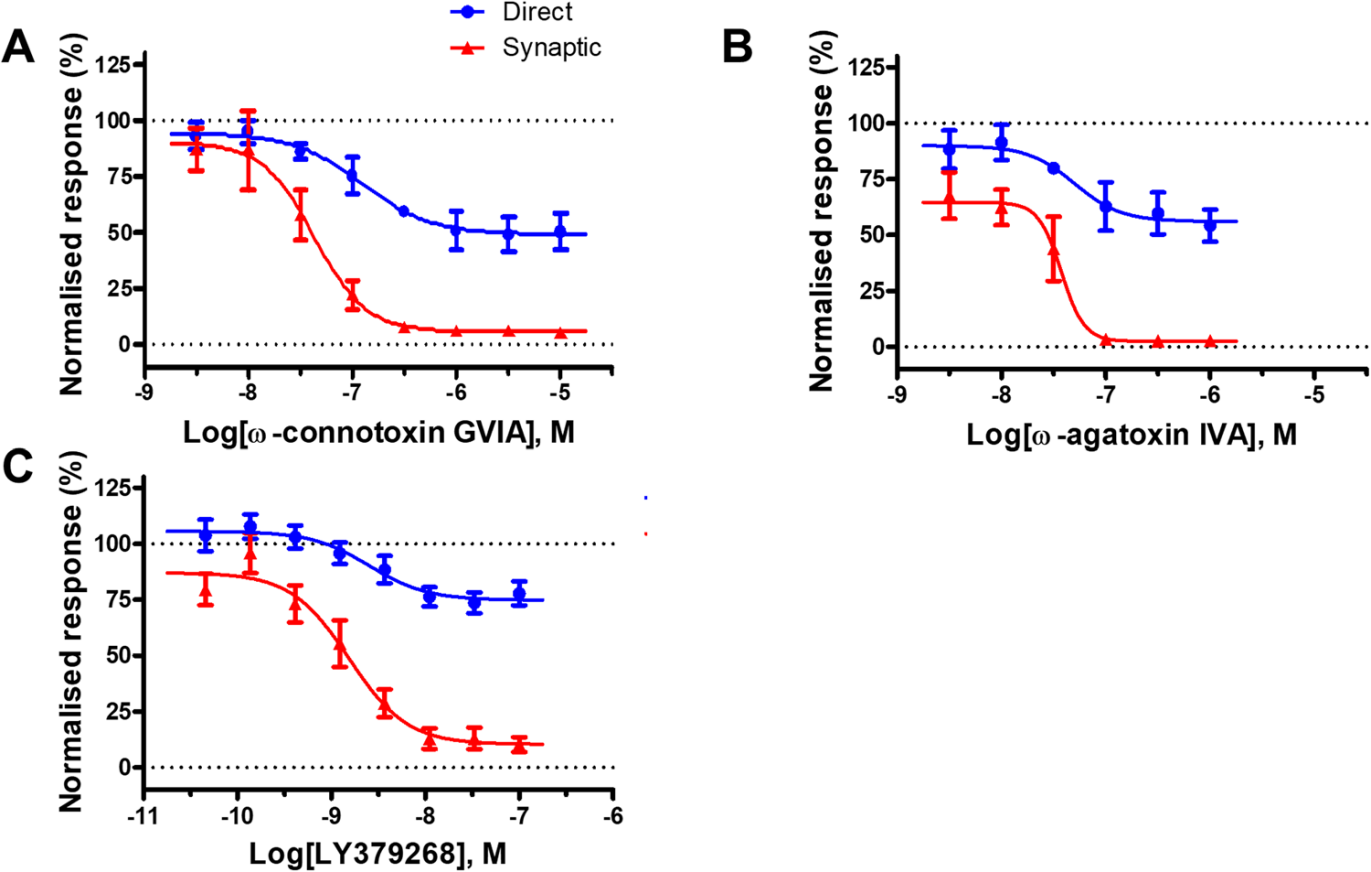
Modulation of EFS evoked calcium responses by compounds acting at presynaptic receptors. (**A**) ω-connotoxin GVIA, a potent Cav2.2 selective blocker, showed a dose dependent decrease in the synaptic response (10 stimuli, 20 V, 25 Hz), with an IC_50_ of 42.9 nM. (**B**) Application of ω-agatoxin IVA, a potent Cav2.1 selective blocker, caused a dose dependent decrease in the synaptic response with an IC_50_ of 37.5 nM. (**C**) A potent selective group II orthosteric agonist LY379268 induced a dose dependent block of synaptic transmission with an EC_50_ value of 1.54 nM. For all compounds tested above note also the partial block in the direct response (blue trace) probably cause by direct inhibition of voltage–gated Ca^2+^ channels.

We next went on to evaluate compounds that modulate ion channels and receptors localised in the presynaptic compartment. VGCCs are known to be the main ion channels involved in neurotransmitter release^23^. We evaluated the effects of two toxins: ω-agatoxin IVA, a potent Cav2.1 selective blocker and ω-connotoxin GVIA, a potent Cav2.2 selective blocker (Fig. 7A and B). Both toxins caused full block of the synaptic responses with IC_50_ values of 37.5 nM for ω-agatoxin and 42.9 nM for ω-connotoxin respectively. The two toxins had also a robust, although partial effect on the amplitude of the direct responses. Because the readout in our assay is based on calcium influx through voltage-gated Ca^2+^ channels we expect that compounds that block Ca^2+^ channels (like ω-agatoxin and ω-connotoxin) will appear active in the direct EFS responses. The level of block was 55.7 ± 6.1% for ω-agatoxin and 49.5 ± 4.8% for ω-connotoxin. So the partial block we have seen with the two toxins on direct EFS responses relates to effects on VGCCs of the corresponding subtype (Cav2.1 or Cav2.2). The potency of inhibiting the direct EFS responses was close to the one seen on the synaptic responses (123.5 nM for ω-connotoxin and 52.2 for ω-agatoxin respectively).

The role of group II metabotropic glutamate receptors (mGluR2 and mGluR3) on modulating EFS evoked responses in the FLIPR assay was also evaluated. We have used here a potent selective group II orthosteric agonist LY379268^24^ and have shown a concentration dependent block of synaptic transmission with an EC_50_ value of 1.54 nM (Fig. 7C). We also observed a partial block (74.8 ± 4.54%) on direct responses with a similar EC_50_ value, 2.54 nM most probably related to indirect inhibition of voltage-gated Ca^2+^ channels.

The specific effects on EFS evoked synaptic responses observed here with different classes of pharmacological modulators targeting pre- and postsynaptic receptors shows the ability of our model to discriminate and measure synaptic transmission in cultured neuronal networks.

## Discussion

We present here the development, characterisation and validation of a novel high-throughput model for assessing neuronal function and synaptic transmission in primary cultured neurons. The model relies on indirect calcium flux measurements of neuronal activity triggered by EFS. The novelty of the approach originates in the implementation of the electrical field stimulation and data collection in spatially separated areas of an interconnected neuronal network cultured in multiwell plates.

We have shown first the ability to record robust EFS evoked calcium responses at the single cell level by using live cell imaging. We demonstrated by using selective pharmacological tools that EFS evoked neuronal activity can propagate throughout the neuronal network developed following long term culture of primary cortical neurons. We have successfully transferred the assay to a high-throughput cellular screening instrument (FLIPR Tetra) and further optimised the conditions of the assay to enable simultaneous multiwell recordings of EFS evoked responses. We have finally performed a systematic pharmacological validation of the model by investigating activity of a series of compounds with a well-established mode of action.

The conventional methods used to investigate neuronal and synaptic function are typically low-throughput electrophysiology techniques (voltage-clamp, current-clamp, field recordings in cultured neurons and/or in acute slice preparation). These methods provide key information for confirming and understanding the mechanism of action of novel compounds, an essential activity in the drug discovery process. High-throughput electrophysiology instruments have been also developed and are used for drug discovery projects but they are usually applied for studying heterologous expressed ion channels^25^ and only sporadically used to investigate ion channels in neuronal preparations^26^, therefore having some limitations.

Alternative optical methods have been developed and allow for increased throughput by performing the experiments in multiwell plates. These methods use fluorescent indicators in the form of organic dyes or genetically encoded sensors and have been combined with electrical field stimulation in order to investigate function and pharmacology of various ion channels^27–29^. These methods use dedicated instruments (usually not commercially available) and have been optimised for recordings performed in stable cell lines overexpressing ion channels (ie Nav, Cav^27^).

More recently another methodology has been reported that uses EFS combined with fluorescent measurements of a genetically encoded sensor (synapto-pHluorin) to assess neurotransmitter vesicle-cycling in cultured primary rodent neurons^10^. The assay provides a robust and reproducible output and has been used successfully to screen small compounds libraries as reported in^10^. One limitation of this model is the exclusive assessment of the presynaptic component of the synaptic transmission. As a result the assay can potentially miss or cannot differentiate between compounds affecting the neuronal excitability versus compounds acting at the postsynaptic compartment. Another limitation of the methodology described in^10^ is the requirement for relatively long EFS trains in order to achieve a good signal to noise ratio (>10 sec stimulation).

The key aspect of the approach we present here is the ability to stimulate and record in spatially separated areas within the same well of a multiwell plate. This can be achieved by using small diameter electrodes that can be freely positioned inside the well. In the format of the assay we describe here the EFS is used to trigger the excitation in the neurons positioned under the electrodes. Once triggered the excitation propagates between the interconnected neurons within the neuronal network through synaptic transmission. The model provides two independent readouts. The first one, recorded from the neurons located under the electrodes, represents a direct measure of neuronal excitability. The second readout, recorded further away from the stimulation area, provides a measure of synaptic transmission corresponding to the propagation of the excitation at the synaptic level throughout the network. The sodium channels blockers (TTX and lamotrigine) or K^+^ channels modulators (Retigabine) we used in this study had a direct impact on the direct readout because they interfere with generation of APs in the stimulated neurons. The potency values we obtained for the activity of these compounds on the direct response are similar to the literature data^27,30^. One interesting and consistent result obtained with all three tested compounds is the shift to the left of the concentation response curve (CRC) for the synaptic responses. We can hypothesize that the shift in potency is related to the nature of the excitation driving generation of APs in the two readouts: in the direct response it is the electrical stimulus (used in our recordings at a supramaximal amplitude) and in the synaptic response it is represented by the neurotransmitter mediated postsynaptic depolarisation (physiological stimulus). Hence, we can state that the compound potency as measured by using the synaptic response is more representative for the activity of the compound at native receptors.

The data we obtained from the evaluation of the compounds acting at postsynaptic receptors (NBQX, GYKI-53784, NVP-AAM007) validated our initial premise and showed distinct effects (full inhibition) on the synaptic function readout versus no effects on the neuronal excitability readout. While we expected to see block of synaptic activity by antagonists of AMPA receptors, which are known to be the main drivers of fast synaptic transmission, we were intrigued about the effect of the NMDA antagonist. NMDA receptors play an essential role in the induction of synaptic plasticity, a phenomenon believed to be the main cellular mechanism underlining learning and memory formation in central nervous system^31–33^. In studies performed in embryonic neurons, similar to the preparation used in this work, it has been shown previously that NMDA receptors can also mediate synaptic transmission^34,35^.

Further validation of the synaptic nature of the assay described here was provided by data obtained with the compounds acting on the presynaptic compartment (Cav blockers and mGluR2/3 agonist). Both Cav toxins used here (ω-agatoxin for Cav2.1 and ω-connotoxin for Cav2.2) caused partial block of somatic calcium responses, as measured in the direct response component. Assuming that a similar level of block has been achieved at the presynaptic level this would probably be enough to cause full block of neurotransmitter vesicle release. Previous studies have shown that neurotransmitter release has a non-linear dependence upon the Ca^2+^ influx^36,37^ so a partial reduction in intracellular calcium at the presynaptic compartment can cause full block of release. The inhibition of the synaptic responses seen with the mGluR2/3 agonist is also related to the effects on voltage-gated Ca^2+^ channels (partial block of the direct response). Activation of the pre-synaptic group II mGlu receptors is known to inhibit neurotransmitter release by modulation of voltage gated Ca^2+^ and K^+^ channels^38–40^.

The selective effects observed here with different classes of neuronal/synaptic function modulators demonstrate the ability of our model to specifically investigate and identify essential components involved in synaptic function.

Pathological changes in synaptic function, structure and number have been associated with various psychiatric and neurological disorders. A dramatic reduction in the synapse number has been observed in post-mortem AD brain tissue^1–3^ which showed a high correlation with cognitive dysfunction^4,5^ suggesting that synaptic changes are crucial for AD pathogenesis. Alterations in neuronal and synaptic function have been also reported for other neurological disorders: schizophrenia^41–43^, autism^44,45^, and epilepsy^46,47^.

Recent breakthroughs in our ability to generate patient-specific induced pluripotent stem cells (iPSCs) through reprogramming of somatic cells^48^ and then to further differentiate them into functional neurons allows us today to study neuronal and synaptic pathophysiology in live human neurons *in vitro* ^49,50^. Great progress has been made recently on establishing and characterisation of novel human IPS neuronal disease models^51–57^. Some of these studies have identified particular functional phenotypes that can in the future be transferred and investigated further by using the synaptic function assay we present in this report.

Screening by using the functional phenotypic assay reported here, in primary rodent neurons under normal conditions, can potentially lead to discovery of compounds with novel mechanism of action and/or identification of novel targets involved in synaptic pathophysiology.

The methodology we described in this study represents a unique phenotypic pharmacological screening model that can be used for investigating synaptic transmission in a high throughput mode. This model has multiple applications in the neuroscience field and can impact directly research and drug discovery projects in the areas of pain, psychiatry and neurodegenerative diseases.
